# Nonreciprocal terahertz beam steering based on magneto-optic metagratings

**DOI:** 10.1038/s41598-019-56789-x

**Published:** 2019-12-27

**Authors:** Zhiyu Tan, Fei Fan, Xipu Dong, Jierong Cheng, Shengjiang Chang

**Affiliations:** 1 Institute of Modern Optics, Nankai University, Tianjin, 300350 China; 2 Tianjin Key Laboratory of Optoelectronic Sensor and Sensing Network Technology, Tianjin, 300350 China

**Keywords:** Metamaterials, Magneto-optics, Terahertz optics

## Abstract

In this work, an active nonreciprocal THz beam steering has been proposed based on a transversely magnetized metal/InSb metagrating. The nonreciprocal dispersion relation and phase shift characteristics of the metal/InSb waveguide are investigated in details. A metagrating structure with gradient phase shift has been designed based on the metal/InSb waveguide. Under the external magnetic field (EMF), the THz beam can be changed among 0, +1^st^, and −1^st^ order of the metagrating. Due to the nonreciprocity of the metal/InSb metagrating, the deflection angle can be controlled by changing the positive and negative directions of the EMF, to realize bilateral symmetric scanning from −67.8° to 67.8° with over 70% diffraction efficiency, and this device also exhibits the nonreciprocal one-way transmission as an isolator with the isolation of 13 dB. This low-loss, large deflection degree, nonreciprocal beam scanner has a great potential application in the THz regime.

## Introduction

Terahertz (THz) techniques in imaging, radar, and communication systems have been greatly developed in recent years^1^. To realize these application systems, THz functional devices, such as THz modulators^2^, isolators^3^, phase shifter^4^, and polarization convertor^5^ are indispensable. Among them, active THz beam steering devices can control the propagation direction of THz waves in the free space, which is the core device in the above application systems, and thus are in highly demand^6^. In recent years, many THz beam steering technologies and devices have been reported, including mechanical scanning^7^, phase array^8^, and frequency scanning antennas^9^, especially reconfigurable metasurfaces based on electrically or optically active semiconductor and 2D materials (*e.g*. silicon and graphene)^10^, phase transition materials (*e.g*. VO_2_)^11^, tunable materials (*e.g*. liquid crystals)^12^, and micro-electromechanical (MEMs) technology^13^.

However, the performances of these devices are still limited in the THz regime. Frist, it is difficult to achieve an active deflection with both high efficiency and large angle (hard to go beyond 60°). For examples, Monnai *et al*.^7^ reported a reconfigurable THz beam steering antenna using programmable micromechanical gratings. The adjustable angle of deflection can be achieved only from −30° to 10° at 0.3 THz. Hashemi *et al*.^11^ reported an electrically controlled beam steering device based on a VO_2_ metasurface can achieve 20° deflection at sub-THz frequencies. An optically tunable metasurface was also developed based on dielectric metasurface and liquid crystal by Komar *et al*.^12^, of which deflection only reach 12°. In addition, due to the reciprocity of wave transmission in the device, the phased array structured by asymmetric gradient metasurface can only deflect beam to one side, so it is difficult to realize bilateral symmetric scanning no matter how to change the intensity or direction of the biased electric field. For example, Perez-Palomino *et al*.^14^ reported a liquid crystal reflective array antenna can deflect THz beam from −5° to −60°, but the positive angles cannot be scanned. Programmable metasurfaces can provide more flexible beam manipulation^15^, but the precision fabrication of such device is still a challenge in the THz regime.

The unique nonreciprocal effect and magnetic tunability of magneto-optical (MO) device make it play an irreplaceable role in the THz functional devices. For examples, our previous works reported THz nonreciprocal isolators based on MO plasmonics and metasurface composed of InSb^16,17^, which can achieve high isolation ratio of over 40 dB. Wang *et al*.^18^ experimentally observed an interference-induced transparency effect in InSb, which shows its good magnetic tunability in the THz regime. Shen’s group proposed the broadband THz one-way transmission based on the MO photonic crystal and MO plasmonic structures^19,20^. Recently, we also experimentally reported a THz nonreciprocal circular dichroism and Faraday effect of over 90° rotation angle under a weak magnetic field^20^, which can be used as THz MO polarization convertor and modulator. Furthermore, the tunable nonreciprocal phase shift property of this material has also been demonstrated. MO materials are commonly used in phased array antennas in the microwave band^21,22^. However, so far, the beam steering devices controlled by the magnetic field have been rarely reported yet in the THz regime. Combining THz MO material within artificial microstructure, magnetically controlled THz beam steering devices are expected.

In this paper, we have proposed active nonreciprocal THz beam steering based on a transversely magnetized metal/InSb metagrating. The nonreciprocal dispersion relations and phase shift characteristics of the metal/InSb waveguide are investigated in detail. On this basis, the metagrating structure with gradient phase shift is designed. Under the external magnetic field (EMF), the main beam can be changed among 0, +1^st^, and −1^st^ order of the metagrating, which reaches a large deflection angle of 67.8° with over 70% diffraction efficiency at the central working frequency of 0.9 THz. More importantly, due to the nonreciprocity of the metal/MO metagrating, the deflection angle can be controlled by changing the positive and negative directions of the EMF, to realize bilateral symmetric scanning from −67.8° to 67.8°, and this device also exhibits the nonreciprocal one-way transmission as an isolator with the isolation of 13 dB.

## Theoretical Analysis

### Magneto-optical property of InSb in the THz regime

In this work, we use the undoped InSb as MO semiconductor material. In the THz regime, when the EMF is along the *z* direction, the dielectric function of InSb becomes a nonreciprocal tensor, which can be expressed as^23–25^:

1 $$\varepsilon =(\begin{array}{ccc}{\varepsilon }_{1} & -i{\varepsilon }_{2} & 0\\ i{\varepsilon }_{2} & {\varepsilon }_{1} & 0\\ 0 & 0 & {\varepsilon }_{3}\end{array})$$

where three different tensor elements in Eq. (1) can be written as:

2 $${\varepsilon }_{1}={\varepsilon }_{\infty }-\frac{{\omega }_{p}^{2}(\omega +\gamma i)}{\omega [{(\omega +\gamma i)}^{2}-{\omega }_{c}^{2}]},{\varepsilon }_{2}=-\frac{{\omega }_{p}^{2}{\omega }_{c}}{\omega [{(\omega +\gamma i)}^{2}-{\omega }_{c}^{2}]},{\varepsilon }_{3}={\varepsilon }_{\infty }-\frac{{\omega }_{p}^{2}}{\omega (\omega +\gamma i)}$$

Here, *ω*_*c*_ is the cyclotron frequency, when an EMF is applied, the semiconductor InSb shows a strong gyrotropy near this frequency. *ω*_*c*_ is proportional to the EMF by *ω*_*c*_ = *eB*/*m*^*∗*^, where *m*^*∗*^ is the effective mass of the carrier, for the InSb, *m*^*∗*^ = 0.014*m*_*e*_*, B* is the magnetic flux density, *m*_*e*_ is the mass of electron, and *e* is the electron charge. *ε*_*∞*_ is the high-frequency limit permittivity, *ε*_*∞*_ = 15.68; *ω* is the circular frequency of the incident THz wave; *ω*_*p*_ is plasma frequency written as *ω*_*p*_ =  (*Ne*^2^/*m*^*∗*^*ε*_0_)^1/2^, where *N* is the carrier density, *ε*_0_ is the free-space permittivity; *γ* is the collision frequency of carriers, *γ* = *e*/(*μm*^*∗*^), and *μ* is the carrier mobility, which is modeled as *μ* = 7.7 × 10^4^ (*T*/300)^−1.66^ cm^2^∙V^−1^∙s^−1^, so the *γ* is also dependent on the temperature.

Moreover, the dielectric property of the InSb greatly depends on the intrinsic carrier density *N*, and the *N* strongly depends on the temperature *T*, which follows^26,27^

3 $$N(c{m}^{-3})=5.76\times {10}^{14}{T}^{1.5}\times \exp [\,-\,0.26/(2\times 8.625\times {10}^{-5}\times T)]$$

The dielectric tensor of the InSb shows a strong dispersion and MO properties, and it is strongly dependent on the EMF and temperature in the THz regime.

### The dispersion of metal/InSb waveguide structure

**Figure 1:**
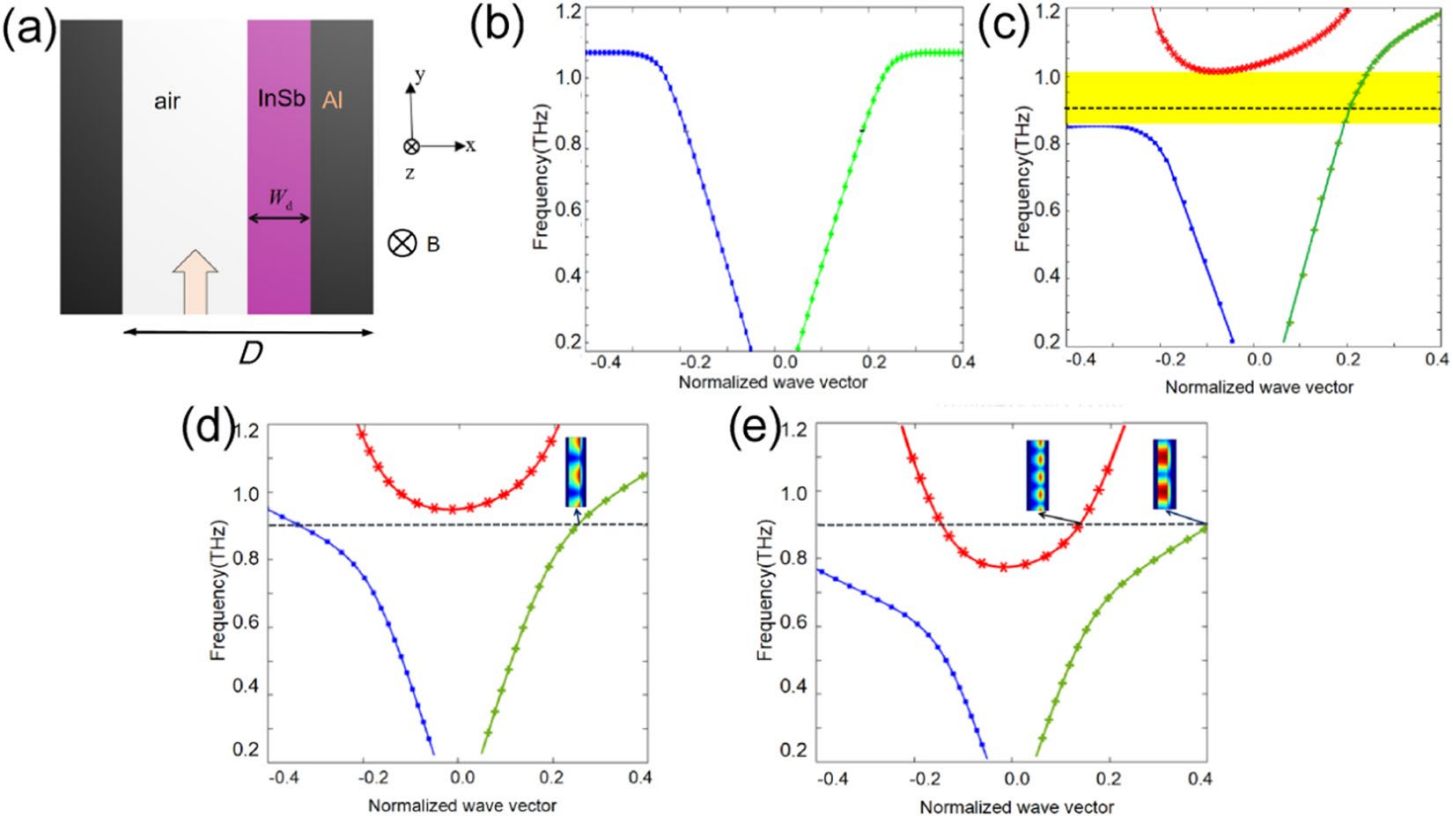
(**a**) Schematic of metal/InSb waveguide; (**b**) the dispersion curves of the waveguide structure when *B* = 0 T and *W*_*d*_ = 20 μm, the *x*-coordinate is the normalized wave vector *k*, where the propagation constant *β* = *k* × 2*π*/a; (**c**) dispersion curves when *B* = 0.5 T and *W*_*d*_ = 20 μm, which shows an isolation band around *f* = 0.9 THz; (**d**) dispersion curves when *B* = 3 T and *W*_*d*_ = 20 μm, which has only one propagation mode at the frequency of 0.9 THz; (**e**) dispersion curves when *B* = 3 T and *W*_*d*_ = 27 μm, at 0.9 THz, which has two different modes.

We designed a metal/InSb waveguide structure to manipulate the nonreciprocal phase shift of THz waves, as shown in Fig. 1(a), The InSb and metallic Al have the same width *W*_*d*_ and the whole width of air/metal/InSb waveguide is *D* = 120 ${\rm{\mu }}{\rm{m}}$. The incident wave is TM polarization (the electric vector is along *x* axis), and the EMF is applied along the + z direction. The vectors of ***K***, ***E*** and ***B*** are orthogonal to each other.

In this configuration, by applying the wave equation *k*^2^*E* − *k*(*k* ∙ *E*) − *ω*^2^*εμ* ∙ *E* = 0 and boundary conditions, the dispersion curves can be obtained in Fig. 1 by numerical simulation with the finite element method (FEM). Here, we use the Floquet period condition along the wave propagation direction, the period is set as *a* = 60 μm, thus the propagation constant *β* = *k* × 2π/*a*, *β* > 0 means forward propagation, and *β* < 0 for backward propagation. The positive and negative propagation constant is equivalent to the positive and negative directions of the EMF because of the nonreciprocity of this MO system. When *B* = 0 T, *T* = 235 K and *W*_*d*_ = 20 μm, the dispersion curves are symmetric as shown in Fig. 1(b). When the EMF is applied, due to the gyrotropy of the InSb and the asymmetric structure, the surface plasmon splits as two different magneto surface plasmons (MSPs) with different dispersion relations. When *B* = 0.5 T, the calculated dispersion curves shown in Fig. 1(c) becomes asymmetric, and there is a one-way transmission band around the frequency of 0.9 THz (yellow range in Fig. 1(c)), where the forward wave can transmit through the waveguide but backward waves are forbidden when the EMF is positive. When the EMF increases to *B = *3 T, the isolation band disappears at 0.9 THz as shown in Fig. 1(d). When the width extends from *W*_*d*_ = 20 μm to 27 μm, the dispersion curves of modes move to lower frequency range, so there are two different modes located at 0.9 THz (the green curve and red curve) shown in the Fig. 1(e). It can be concluded that the mode dispersion relations of metal/InSb waveguides vary greatly with different value and direction of EMF, and with different widths of waveguides, which leads to changes in the phase shift characteristic of the output wave.

### Design

**Figure 2:**
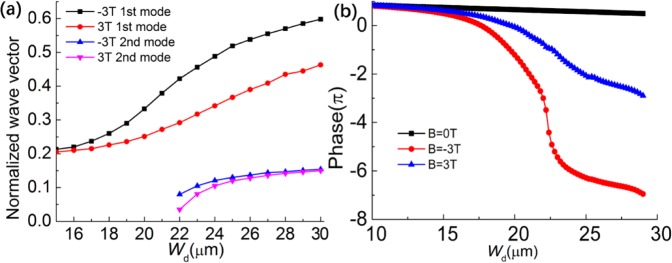
(**a**) Normalized wave vector as the function of *W*_*d*_ at 0.9 THz, which shows the difference between the positive and negative EMF. (**b**) phase shift as a function *W*_*d*_ at 235 K of these proposed unit cells in different EMF: *B* = 0 T, −3T and 3 T.

We further discuss the relationship between the normalized wave vector *k* and *W*_*d*_ at 0.9 THz as shown in Fig. 2(a). We increase the *W*_*d*_ when the width of the waveguide *D* remains the same, and so the width of air decreases. The results demonstrate that the *k* increases with *W*_*d*_. At the same frequency, a larger wave vector indicates a larger phase shift of mode, so the phase shift of output waves changes with the width of metal/InSb. More importantly, the wave vectors of negative EMF increase faster than that of the positive one, which indicates the quite difference of phase shift of this metal/InSb waveguide when we apply positive and negative EMF. The phase shift of metal/InSb waveguide as a function of *W*_*d*_ under *B* = 0, ±3 T at 0.9 THz are simulated by the finite element methods (FEM). As shown in Fig. 2(b), the output phase delay is increasing with the width growing of metal/InSb bar. When the magnetic field of ±3 T is applied, the phase changes faster than that of 0 T. And due to the non-symmetry of the dispersion curves we discussed above, the phase shift is quite different between positive and negative directions of EMF as shown in Fig. 2(a).

**Figure 3:**
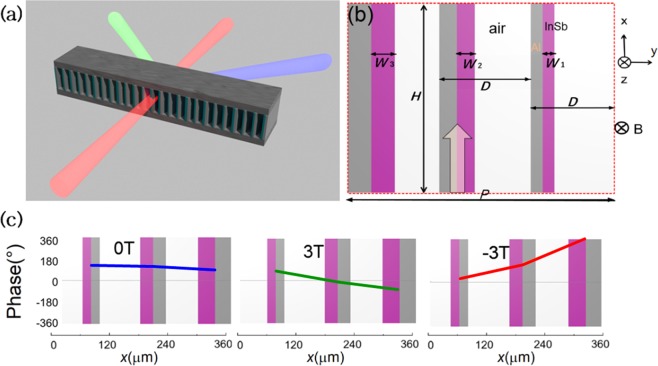
(**a**) Schematic of the metal/MO metagrating for tunable beam steering. (**b**) Side view of one proposed supercell, *H* = 0.48 mm.*W*_1_, *W*_2_, and *W*_3_ are width of three different InSb or metal bar in the waveguide, respectively. Each waveguide has the same total width *D* = 120 μm. (**c**) Schematic diagrams of phase distribution at the proposed supercell when *B* = 0T, −3T, 3T respectively. Here, the phase is constraint in the range of −360–360°.

Based on the above discussion, by designing metal/InSb waveguides of different *W*_*d*_ to form a gradient phased array, the directional beam can be modulated on the different diffraction order with different deflection angles under the different EMF. This one directional subwavelength gradient meta/InSb array in the form of grating also can be called as the meta-grating^28,29^. The schematic of this proposed MO metagrating is shown in Fig. 3. The metagrating consists of several supercells, and there are three different metal/InSb waveguides with different width *W*_*d*_ of metal/InSb bar in one supercell as shown in Fig. 3(b). The width of each waveguide is still *D* = 120 μm, the period of one supercell is *P* = 360 μm, and the length of this device is *H* = 0.48 mm.

To realize the nonreciprocal beam steering, we choose three waveguide as sub unit cell with the different *W*_*d*_ of 17 μm, 20 μm and 27 μm to form a supercell. The phase distribution of the proposed supercell under 0T, 3T and −3T is shown in Fig. 3(c), respectively. When *B* = 0T, there’s only a slight phase difference in three unit cells, thus the output beam is converged on 0 order of metagrating. When *B* = −3T, the phase increases from *W*_1_ to *W*_3_, but when *B* = 3T, the phase decreases from *W*_1_ to *W*_3_. By appropriately adjusting the width of each meal/InSb bar to form a gradient phased array, the directional beam can be modulated on the different diffraction order with different deflection angles.

## Result and Discussion

**Figure 4:**
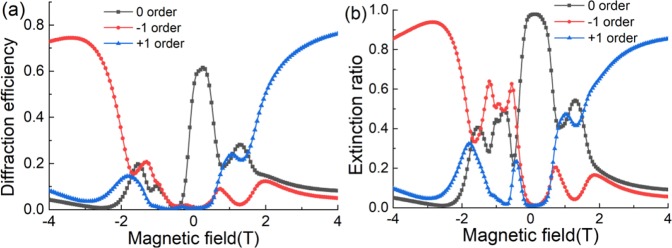
(**a**) Diffraction efficiency curves of different diffraction orders discussed above under different EMF. (**b**) Extinction of different diffraction orders in proportion to the total transmittance.

To verify the above analysis, we simulate the diffraction property of this proposed metagrating. Because the period of the proposed supercell *λ* > *D* > 2*λ*, there are only 0 and ±1^st^ orders at 0.9 THz. First, the diffraction efficiency of three diffractive orders *v.s*. the EMF is calculated by using the rigorous coupled-wave analysis (RCWA) method as shown in Fig. 4(a). The extinction ratio which is defined as the diffraction efficiency in proportion to the total intensity transmittance, *i.e. E*_*xt*_ = *T*_*n*_/*T*_*total*_, is shown in Fig. 4(b). When *B* = 0T, there is no MO effect, the output beam is mainly on the 0 order with the efficiency of 68%, and the extinction ratio is near to 100%. As the EMF increase, the diffraction efficiency of 0 order decrease quickly due to the MO effect. And for the positive and negative EMF, the changes are different: when *B* = −0.5T, the diffraction efficiency of all diffractive orders tends to be 0, but under the positive EMF, the transmittance is still 50%, which shows the nonreciprocal isolation effect. With the efficiency of 0 order decrease, the diffraction efficiency for ±1^st^ order increasing, and it is also different for the different directions of EMF: when *B* > 2T, the diffraction efficiency for +1^st^ order increase quickly, but the efficiency of 0 and −1^st^ orders tend to drop off, thus the extinction ratio also increase with the EMF, when the EMF increase to +3 T, the efficiency for +1^st^ order is 72% with the extinction ratio of 85%; But for the negative EMF, it has the same trend for −1^st^ order, the efficiency for −1^st^ order is increasing with the negative EMF, when *B* = −3T, the diffraction efficiency for −1^st^ order reaches 74% and the extinction ratio is 92%.

It is noted that most of metasurfaces for beam steering or deflection are typically suffering from the issue of low efficiency in the large diffraction angle, extremely for the transmitted structure. Due to the undesired coupling between the neighboring elements, the local phase response of the element in the gradient array is approximated by its response in the uniform array, making it quite difficult to obtain desired gradient phase, so it is difficult to modulate the energy as much as possible from zero order to the higher diffractive order with large diffraction angle^29,30^. Here, this issue is well solved by the developed metal/InSb meta-grating. The coupling effect between neighboring unit cells can be eliminated because of the isolation of the metallic boundaries in each unit cell, so that the large gradient phase could be obtained by changing the width of metal/InSb waveguide element under the EMF. Meanwhile, the nonreciprocity of metal/InSb waveguide structure can enhance the magneto-optic effect and reduce the return loss. These factors synthetically determine the large deflection angle with relatively high diffraction efficiency.

**Figure 5:**
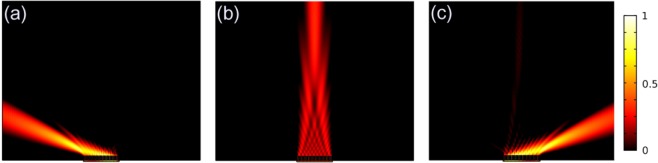
Field distribution of the metal/MO metagrating, when the 0.9 THz wave normally incident from the port, the output beam can be tuned on the different diffractive orders: (**a**) *B* = −3T, wave output vertically for −1^st^ order; (**b**) *B* = 0 T, wave tuned to 0 order; (**c**) *B* = 3 T, wave tuned to +1^st^ order. For 1st order, the deflection angle is 67.8°.

Then we focus on the spatial power flux distribution of the 0.9 THz wave transmitting through this metagrating under 0 T, 3 T, −3T as shown in Fig. 5(a–c), respectively. Here, the deflecting angle *θ* = ±67.8° for ±1^st^ order is determined by the grating equation *P*sin*θ* = *λ*. As we can see, by varying the magnetic field, this device can realize the beam steering, and for the positive and negative direction of the EMF, the deflection angle of the output wave is opposite.

**Figure 6:**
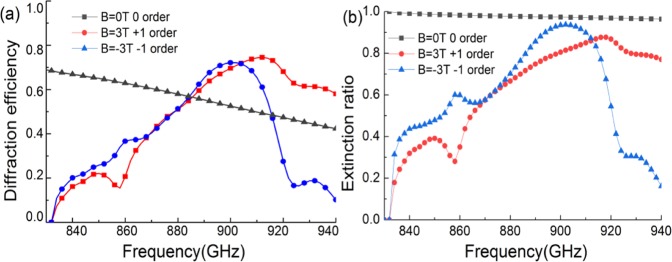
(**a**) Diffraction spectra of the supercell for different diffractive orders at the corresponding EMF. (**b**) Extinction ratio of these diffractive orders.

We further discuss the frequency response, we calculate the diffraction efficiency under some specific EMF, *i.e. B* = 0 T, −3 T, and 3 T. The diffraction efficiency is changed due to the output phase distribution is varied with the frequency. When *B* = 0 T, the output wave is always modulated on the 0 order as there is no MO effect within the device, so we focus on the efficiency of 0 order. As the black curve shown in Fig. 6(a), the diffraction efficiency decreases when the frequency rises up, but the extinction ratio changes slightly as shown in Fig. 6(b). When *B* = 3 T and −3T, we mainly focus on the ±1^st^ order diffractions as the red line and blue line shown in Fig. 6(a,b). Because of the diffraction equation, the ±1^st^ order diffractions only appear in the frequency range of *f* > 833 GHz. At first, the diffraction efficiency of ±1^st^ order increases with the frequency, but when the frequency is over 0.9 THz, it decreases gradually. For ±1^st^ order, the extinction ratio has the same trend as the diffraction efficiency curves. The bandwidth of this device is around 0.88–0.91 THz, where the efficiency is more than 40% and the extinction ratio is more than 60%.

**Figure 7:**
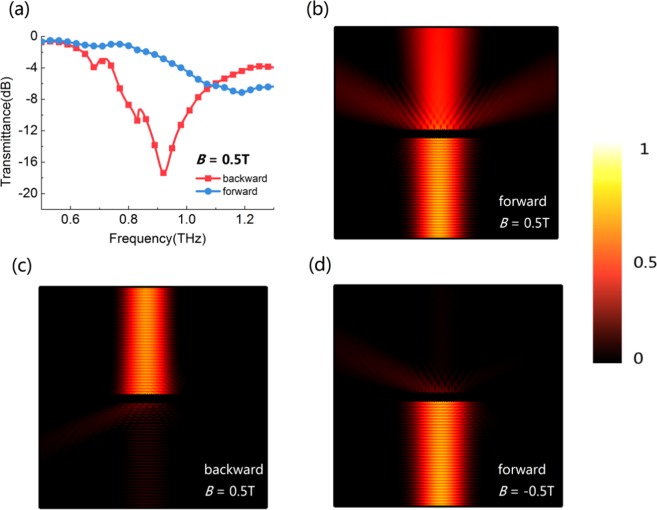
(**a**) Transmittance of the forward and backward beam under the EMF of *B* = 0.5 T. The field distribution in different conditions: (**b**) forward beam, *B* = 0.5 T; (**c**) backward beam, *B* = 0.5 T; (**d**) forward beam, *B* = −0.5 T.

Moreover, due to the nonreciprocity that has discussed above, we also take attention to the isolation effect in this device. As shown in Fig. 7(a), the transmission spectra of forward and backward waves are simulated, for which exhibiting one-way transmission effect. When *B* = 0.5 T and *f* = 0.9 THz, the transmittance of forward beam is more than −3 dB, but for the backward beam, the transmittance is less than −16 dB. The field distribution is also simulated in Fig. 7(b–d). When *B* = 0.5 T, the forward beam can pass through the metagrating, but the backward beam cannot. When the magnetic field reverses, the opposite case happens. The results of changing the propagation direction and the direction of magnetic field are equivalent, as shown in Fig. 7(c,d), which are the typical characteristics of magneto-optical nonreciprocal transmission. In a word, the device works as an isolator of nonreciprocal one-way transmission with an isolation of 13 dB at 0.9 THz when *B* = 0.5 T.

**Figure 8:**
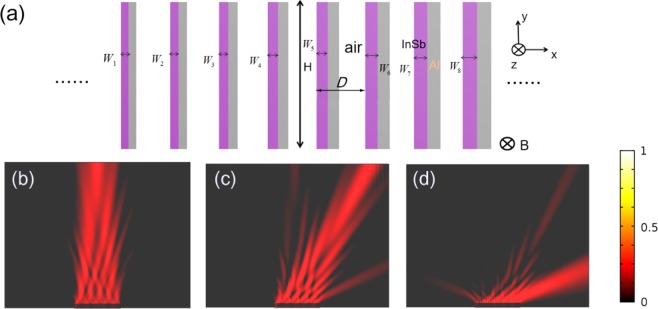
(**a**) Side view of proposed 8-period supercell including external magnetic field and coordinate system in this work, *H* = 0.3 mm and *D* = 80 μm. The field distribution of the diffraction is shown, by this proposed 8-period metagrating, we can realize 3-angle sweeping by different EMF: (**b**) *B* = 0 T for 0 order output; *B* = 2 T; (**c**) the wave is tuned to +1^st^ order, for 1^st^ order diffraction, the diffraction angle is 30°; (**d**) *B* = −5T for +2^nd^ order output, the diffraction angle is 69.6°.

At last, by using the same design method, we can also realize the 2^nd^ order sweeping by designing an 8-period gradient metagrating as shown in Fig. 8(a), the period of the unit cell is *D* = 80 μm, the whole width of one supercell is *P* = 640 μm, and the length of this metagrating is *H* = 0.3 mm. When the frequency is 1 THz, the temperature *T* = 240 K, this proposed metagrating can steer the diffractive orders to 0, +1^st^ and +2^nd^ orders under different EMF as shown in Fig. 8(b–d), respectively. When *B* = 0 T, the beam is on the 0 order with the efficiency of 55%; when *B* = 2 T, the wave is tuned to +1^st^ order with the efficiency of 31%, the diffraction angle is 30°; when *B* = 5 T, the output wave is tuned to +2^nd^ order with the efficiency of 45%, and the diffraction angle is 69.6°.

## Conclusion

In summary, due to the special dispersion and magneto-optical property of the InSb, the asymmetric dispersion relation and tunable diffraction property of the proposed metagrating are investigated in the THz regime. The numerical results show that the metal/MO metagrating can steer THz waves to the different diffractive orders by changing the EMF. The diffraction efficiency is more than 68% with an extinction ratio of over 85% at 0.9 THz. Because of the nonreciprocity of this device, the deflection angle can be controlled by only changing the positive and negative directions of the EMF, so it can realize bilateral symmetric scanning from −67.8° to 67.8°. In addition, this device also can be worked as an isolator in the case of *B* = 0.5 T. At last, the 2^nd^ order tuning in an 8-period metal/MO metagrating is also demonstrated. This low-loss, active, nonreciprocal beam scanner has a great potential application in the THz regime.
